# Correction: Diffusion equation for the longitudinal spectral diffusion: the case of the RIDME experiment

**DOI:** 10.1039/d4cp90171a

**Published:** 2024-10-14

**Authors:** Sergei Kuzin, Gunnar Jeschke, Maxim Yulikov

**Affiliations:** a ETH Zürich, Department of Chemistry and Applied Bioscience, Laboratory of Physical Chemistry Vladimir-Prelog-Weg 2 8093 Zürich Switzerland sergei.kuzin@phys.chem.ethz.ch maxim.yulikov@phys.chem.ethz.ch

## Abstract

Correction for ‘Diffusion equation for the longitudinal spectral diffusion: the case of the RIDME experiment’ by Sergei Kuzin *et al.*, *Phys. Chem. Chem. Phys.*, 2022, **24**, 23517–23531, https://doi.org/10.1039/D2CP03039J.

The authors would like to correct an error in the numeric value presented in eqn (4) of the published manuscript. The correct information is presented below:

In eqn (4) the numeric value for the coefficient *B* was incorrectly computed. The correct value is 0.222 MHz nm^3^, four times larger than the value 0.0555 MHz nm^3^ given in the publication. This shifts the effective blocking radius *R*_b_ determined in the model closer to the equality radius *R*_eq_ but does not substantially affect other calculations and discussions. The new value for the effective blocking radius for the fully protonated sample is *R*_b_ = 13.6 ± 0.3 Å. This correction does not affect any fitted *σ* values, as they are determined directly from the fit of experimental data to eqn (16) without involving the blocking radius.

The Royal Society of Chemistry apologises for these errors and any consequent inconvenience to authors and readers.

